
JOURNAL INFORMATION
==============================
NLM Title Abbreviation: Sci China Life Sci
Journal ID: Sci China Life Sci
NLM Journal ID: 101529880

Science China. Life Sciences

ISSN: 1674-7305
EISSN: 1869-1889

ARTICLE INFORMATION
==============================
PMCID: PMC7088909
PMID: 23015132
DOI: 10.1007/s11427-012-4371-2
Article ID: 4371
Article version: 1
Subjects: Insight

A glimpse of enzymology within the idea of systems

Liu ChuanPeng liucp74@hotmail.com
1
Fan DongJie 2
Shi Yi 3
Zhou QiMing 4
1 grid.19373.3f 0000000101933564 School of Life Science and Technology, Harbin Institute of Technology, Harbin, 150080 China
2 grid.198530.6 0000000088032373 National Institute for Communicable Disease Control and Prevention, Chinese Center for Disease Control and Prevention, Beijing, 102206 China
3 grid.9227.e 0000000119573309 Center for Stem Cell and Regenerative Medicine, Shanghai Advanced Research Institute, Chinese Academy of Sciences, Shanghai, 201203 China
4 grid.9227.e 0000000119573309 State Key Laboratory of Mycology, Institute of Microbiology, Chinese Academy of Sciences, Beijing, 100101 China
Electronic publication date: 2012 Sep 27
Print publication date: 2012
Volume: 55
Issue: 9
First page: 826
Last page: 833
Received 2012 Jun 12; Accepted 2012 Jul 21
Copyright: © The Author(s) 2012
License: Open AccessThis article is distributed under the terms of the Creative Commons Attribution 2.0 International License (https://creativecommons.org/licenses/by/2.0), which permits unrestricted use, distribution, and reproduction in any medium, provided the original work is properly cited.
License URL: https://creativecommons.org/licenses/by/2.0/

Keywords: Coagulation Factor VIII, Acetone Cyanohydrin, Curr Opin Chem Biol, Antiviral Innate Immune Response, Deubiquitinase Activity
issue-copyright-statement: © Science in China Press and Springer-Verlag GmbH 2012

==============================

Next year will mark the 100th anniversary of the Michaelis-Menten equation [1], one of the most important and well known models in enzymology. Remarkable progress in enzymology over the past hundred years has not only provided deep insight into biological processes, but also has dramatically changed our lives. However, because of limitations in available techniques, enzymologists primarily investigate the *in vitro* properties and catalytic mechanisms of individual enzymes. Thus, they often fail to address a fundamental scientific question: how are biological processes, most of which are chemical reactions catalyzed by enzymes, coordinated in space and time to produce a living organism [2]? Fortunately, applications of new techniques and, in particular, wide acceptance of the idea of systems have enabled enzymologists to begin studying enzymes in the context of dynamic, complicated biological systems in recent years. Many excellent reviews and research articles on enzymology have been published in the past few years. These articles highlight the latest developments in enzymology, and should prove helpful for understanding how to best integrate the idea of systems into enzymology research.

In living organisms, protein post-translational modification (PTM) pathways are large and interconnected networks. Via reversible or irreversible covalent modifications, allosteric regulations of PTM enzymes and cross-talk of PTM pathways, PTM networks enable organisms to respond to stimuli faster than is possible through gene transcription regulation. Therefore, PTM enzymes, as key nodes of biological networks, are a recent focus of attention.

Phosphorylation is often argued to be the most abundant PTM, and also is the most extensively studied because of the essential contribution of phosphorylation-dependent interaction networks to cellular regulation [3]. In the review by Han *et al*. [4], a novel receptor-like kinase subfamily in angiosperm, CrRLK1-L subfamily, was described in detail. After discussing the structure, enzymological properties and biological function of CrRLK1-L, the authors argued that CrRLK1-L could initiate signal transduction largely independent of the known signaling pathways. However, a better understanding of its allosteric regulation and substrate specificity are still necessary for defining the pathways that it may modulate. Unlike CrRLK1-L, calcineurin is an extensively studied serine-threonine protein phosphatase because it plays a pivotal role in a wide series of crucial physiological processes. Tu *et al*. [5] reviewed the recent literature on a new family of calcineurin regulators (RCANs), and discussed the molecular mechanism for RCAN-mediated regulation of calcineurin. After exemplifying the roles of RCANs in a wide variety of diseases including Alzheimer's disease, the authors suggested that RCANs are potential targets for drug development. Similar to RCANs, glycogen

†Contributed equally to this work
*Corresponding author (email: liucp74@hotmail.com)

synthase kinase-3 (GSK-3) is also involved in Alzheimer's disease, but the underlying mechanism remains unclear. Recently, a research article by Ding *et al.* [6] provided a clue to this question. In their article, one member of SR protein family, 9G8, was identified as a new substrate protein of GSK-3. Hence, the authors suggested that GSK-3 could inhibit 9G8-mediated alternative splicing of tau exon 10 by phosphorylating 9G8, consequently inhibiting the aggregation of tau exon 10.

Ubiquitination is another common PTM. By attachment of ubiquitin to the protein as a monomer or as polyubiquitin chains with different ubiquitin-ubiquitin linkages, the function and fate of the modified substrate can be drastically changed. Ubiquitination is a reversible process regulated by a subtle balance of ubiquitin-modifying enzymes and deubiquitinating enzymes. Two reviews and one research article were recently published, both the review by Xie *et al.* [7] and the research article by Sun *et al.* [8] focused on the deubiquitinating enzymes. Xie *et al.* provided an overview of the recent advances in structural and functional studies of ubiquitin C-terminal hydrolase L1 (UCH-L1), with a special emphasis on the protective effects of UCH-L1 on neurons. Sun and coworkers focused on nsp3, a newly-identified deubiquitinase from human coronavirus NL63. Nsp3 has two papain-like protease (PLP) core domains, and has both protease and deubiquitinase activities. By *in vitro* and *in vivo* methods, the authors demonstrated that the deubiquitinase site was located in the second PLP domain and shared two catalytic residues with the protease site. Furthermore, the authors found the deubiquitinase activity of Nsp3 could induce the deubiquitination of RIG-I and ERIS and then regulate antiviral innate immune responses by inhibition of both RIG-I and TLR3-dependent induction of interferon. Although ubiquitination in eukaryotic systems has been studied for decades, the prokaryotic analog of ubiquitin, ubiquitin-like protein (Pup) was not identified until 2008. Wang *et al.* [9] reviewed the latest advances in research of the Pup-proteasome system and comprehensively discussed its catalytic and regulatory mechanism. The authors suggested that the Pup-proteasome system could be regarded as a potential therapeutic target because of its extensive involvement in many important physiological pathways of pathogenic bacteria.

Acetylation and methylation of histones play fundamental roles in gene expression following environmental stimuli. While acetylation of histones converts chromatin from a closed to an open conformation that facilitates transcription, histone methylation can keep chromatin in either an activated or a repressed state, depending on the precise methylation site and the degree of modification. Interestingly, the intense cross-talk between histone modifications drives distinct downstream functions. Two recent reviews summarize the latest advances of research into two histone modifying enzymes. In the review by Guo [10], the structure and function of a new histone trimethylase, PRDM9 (PR domain containing 9) are described. PRDM9 catalyzes the H3K4 trimethylation, and possesses the activity of a transcription factor and presumably plays an important role in the initiation of recombination. This review strengthens our understanding of species evolution and genetic recombination. Peng *et al.* [11] introduced the latest advances of research into sirtuin proteins. Sirtuins are a highly conserved family of $NAD^+$-dependent deacetylase enzymes and are involved in many important biological processes, including cellular stress resistance, genomic stability, tumorigenesis and energy metabolism. In this review, the authors emphasized the structure and catalytic mechanism of sirtuins and discussed the relationship between sirtuins and aging.

Oxidation of side groups is a very important PTM for cellular proteins. Although Cys and Met are the residues prone to oxidative modification, other residues also can be oxidized under more stringent conditions. For example, the ε-amino group of lysine may be oxidized to an aldehyde by lysyl oxidases (LOXs). LOXs are a family of multi-functional enzymes, and play an essential role in cell proliferation, cell chemotactic responses and tumor genesis. In their review, Zhang *et al.* [12] summarized the latest knowledge of LOXs, including their potential as targets for diagnosis and therapy of several human diseases such as connective tissue disease, exfoliation syndrome, disorders of copper metabolism, pelvic organ prolapse, and bone disorders.

Proteolysis is another one of the most common PTMs. Proteolysis needs to be very precisely controlled on multiple levels because of its irreversible nature. Therefore, enzymes involved in proteolysis are regarded as key nodes in the living system, and have received increasing attention over the past decade. In eukaryotic cells, proteasome-dependent degradation is the general mode of selective protein degradation. While the ubiquitin-dependent proteasomal degradation pathway has been well studied, little attention has been directed toward the possible involvement of the proteasome in ubiquitin-independent proteolysis. However, increasing evidence shows that the ubiquitin-independent proteasomal proteolysis pathway is involved in the elimination of some short-lived regulated proteins, misfolded proteins, aged proteins and oxidized proteins as well as providing "quality control" of newly synthesized proteins. Thus, it plays important roles in pathological processes such as cancer and neurodegenerative diseases. Recent progress in understanding ubiquitin-independent proteasomal degradation and the underlying molecular mechanisms were discussed by Chen *et al.* [13]. Besides the proteasome, proteases also catalyze protein proteolysis. In a review by Wu *et al.* [14], a newly-identified protease, TMPRSS6, was described. TMPRSS6 is a transmembrane serine protease which can regulate the expression of hepcidin, thereby affecting iron metabolism. In this review, the authors discussed the relationship between the structure and function of TMPRSS6, and the roles of TMPRSS6 in iron metabolism-related diseases.

Because proteases are involved in many diseases [15], protease inhibitors have been of great interest for the development of novel therapies. In a recent research article, Tian and coworkers [16] investigated the protease inhibitory activity and antineoplastic effect of buckwheat trypsin inhibitor (BTI). The authors not only identified the active site of BTI, but also obtained two BTI mutants which showed much better stability to heat and pH than wild-type BTI. Their results will contribute to clarifying the physiological mechanism of BTI and support the development of novel anticancer drugs.

Many biologists are now using "omics" tools to investigate organisms and construct various interaction networks. However, because biological systems are essentially built of highly coordinated sets of interaction networks across different scales, integration of the networks from the various 'omics' tools is a core paradigm for deep comprehension and ultimately reconstruction of these living systems. While PTM enzymes act as key nodes in protein-protein interaction networks as discussed above, the enzymes whose substrates are something other than proteins, can bridge protein networks and other networks together. Therefore, the enzymes acting on non-protein substrates are of increasing interest in the systems biology era. Glutamate synthase, lactate dehydrogenase (LDH) and dihydrofolate reductase (DHFR) are three important metabolism enzymes, and play key roles in metabolomics and proteomics data integration. Although these enzymes had been extensively studied, the results presented in three recently published articles indicate that their biological functions are still to be clarified. In an article by Lu *et al.* [17], the authors investigated the roles of glutamate synthase in carbon and nitrogen metabolism of rice, and concluded that glutamate synthase was indispensable for efficient nitrogen assimilation in rice. Meanwhile, new physiological functions of DHFR and LDH were discussed. While Sun and coworkers [18] found DHFR was essential for the development of pharyngeal arches, Li and coworkers [19] suggested that the reduction or loss of LDH-C4 activity in sperm might cause male infertility. These results indicate that our knowledge of "old" enzymes is not complete. Based on the accumulated experiment data on metabolism enzymes, a general relationship between genomics, proteomics and metabolomics of microbes was discussed recently [20,21].

Phase II drug/xenobiotic metabolizing enzymes, together with Phase I enzymes, play a central role in the metabolism and detoxification of numerous exogenous and endogenous compounds. Therefore, the characterization of xenobiotic metabolizing enzymes not only contributes to the understanding of the context of biological networks within organisms, but also is indispensable to elucidate the underlying mechanisms of the interplay between organisms and the environment. Of particular importance is to establish the correlation between the genetic polymorphisms of xenobiotic metabolizing enzymes and interindividual variations in drug metabolism [22]. Hao *et al.* [23] investigated the potential effect of all the known nonsynonymous single nucleotide polymorphisms (nsSNPs) of human phase II metabolizing enzyme on protein function, and identified many residues that are likely to be functionally critical, but have not yet been studied experimentally. Their results contribute to integrating polymorphisms into a physiologically based pharmacokinetic (PBPK) modeling framework.

Since DNA is the carrier of genetic information, the maintenance of DNA stability is essential in living cells. In two recent research articles, the inhibitory mechanisms of helicase and integrase were discussed. Luo and coworkers [24] extensively investigated the effect of lomefloxacin on bloom syndrome helicase (BLM). BLM is an important member of the RecQ family of DNA helicases and plays important roles in DNA repair, recombination, transcription and telomere maintenance. The authors found that lomefloxacin could promote the binding of BLM to DNA, but would unexpectedly inhibit the helicase and ATPase activities of BLM. Subsequently, the authors proposed a possible mechanism based on their kinetics and thermodynamics results which should prove useful for the design of inhibitors against DNA helicase and improve our understanding of the mechanism of action of quinolone drugs. While Luo *et al.* used *in vitro* methods to study BLM, Hu and coworkers [25] investigated the inhibitory mechanism of compound L708, 906 towards HIV-1 integrase by using *in silico* methods. They found the binding of L708, 906 to HIV-1 integrase could alter the mobility of the protein, and thereby result in the decrease of integrase activity. Their results should be helpful for anti-HIV drug design. However, *in silico* results ultimately have to be supported by "real" experimental data. High-throughput screening assays with adequate sensitivity, reproducibility, and accuracy have been indispensable for the discovery of drugs targeting HIV-1 integrase. In a recent research article, He and coworkers [26] developed a novel method for measuring the disintegration activity of HIV-1 integrase. This method is rapid, sensitive and, most importantly, readily adapted to a high-throughput format, and hence is likely to be applicable to antiviral drug screening.

As many chemical and physical factors can cause DNA changes, multiple mechanisms have evolved to deal with DNA lesions in living cells. Translesion synthesis is a special pathway that enables the replication fork to bypass blocking lesions. As a key member of the translesion DNA synthesis pathway, DNA synthesis polymerase kappa (Polκ) has received much attention. In a recent review, Lu *et al.* [27] comprehensively described the structure, catalytic mechanism and biological function of Polκ, which contributes to our understanding of translesion DNA synthesis.

Homologous recombination repair is another powerful DNA repair system. Although homologous recombination has been studied for decades, the underlying mechanism is not yet clear. Since the resolution of holliday-junction recombination intermediates is the critical step for homologous recombination, unraveling the catalytic mechanism of resolvase will contribute to our understanding of homologous recombination. The resolvase from bacteriophage, T7 endonuclease I, is one of the most studied resolvases, and possesses resolvase, nicking enzyme and endonuclease activities. T7 endonuclease I is a homodimer comprising two identical active domains, each of which consists of parts of both subunits in the dimer. However, the function of each active domain is unclear. In a recent research article, Fan and coworkers [28] prepared and characterized a T7 endonuclease I mutant with only a single active domain. Their results showed that the T7 endonuclease I mutant with a single active domain could recognize the holliday-junction structure but failed to introduce two nicked sites simultaneously. Taken together, this information is helpful for elucidating the catalytic mechanism of resolvase.

While many biologists focus on biological networks in cells, tissues and organisms, environmental enzymologists are striving to explore the dynamic nature of ecosystems at the molecular level. The application of meta-omics and enzymology technologies has rapidly increased our knowledge of the global biogeochemical cycles of inorganic and organic compounds, organism-environment and organism-organism interactions. The convergence between enzymology and the ecology of the nitrogen cycle was discussed in recent reviews [29,30]. Zhang *et al.* [31] investigated the phosphorus uptake of *Brassica napus* under low phosphorus stress using enzymological methods, and proposed an acid phosphatase-centric regulatory mechanism of phosphorus uptake. Their results increase our understanding of the mechanisms of the phosphorus cycle. With increasing use of transgenic crops, more scientists are concerned about the environmental impact of transgenic organisms. In a recent research article, Guo and coworkers [32] examined the influence of transgenic Bt cotton on beet armyworm, which is not an insect which targets currently commercialized transgenic Bt cotton varieties in China. Their results show that feeding beet armyworm with Bt cotton can cause significant changes of many important enzyme activities in the worm making it necessary to evaluate the environmental impact of Bt cotton.

While molecular level research on biological systems has evolved from investigating individual enzymes or pathways to studies on biological networks and even ecological networks, a new challenge for biologists is to add a dynamic property to the constructed networks. One strategy is to create an animation to display the dynamic change of biological networks using a series of snapshots of the biological networks captured by high-throughput methods at different points in time. However, because of technical limitations, it is impossible to capture the snapshots at a rate faster than the biochemical reactions, indicating that the "dynamic change of networks" provided by this strategy is actually discontinuous in time, and the biochemical events occurring between any two adjacent snapshots are missed. A better strategy is to incorporate biochemical reaction parameters into biological networks, by which biologists can construct the dynamic biological networks with more "living" properties. Therefore, enzymological studies are crucial not only for the construction of biological networks as discussed above, but also for the elucidation of the dynamical properties of biological systems.

Although enzyme catalysis has been extensively studied, most of this knowledge comes from *in vitro* experiments under conditions in which substrate and product concentrations, mass transfer and diffusion are radically different from the living system. In addition, while canonical kinetic methods provide only ensemble-averaged molecular information, the conformational heterogeneity of an enzyme cannot be ignored in living cells because of the low abundance of enzymes, macromolecular crowding and confinement. Therefore, characterization of enzymes in the context of a biological system is the prerequisite for understanding its dynamic properties. Fortunately, thanks to the close collaboration of scientists and engineers from various disciplines, many novel techniques have recently been developed and applied to the investigation of the *in vivo* behavior of enzymes. For example, mass spectrometry based enzymatic assay techniques have been applied to inhibitor screening *in vivo*, identification of endogenous substrates, and *in vivo* enzymatic activities of orphan proteins [33]. Light and fluorescence microscopy techniques are being used in measuring enzyme activity in single cells in a real-time manner [34,35]. In particular, the development of single molecule detection techniques has made it possible to characterize enzyme catalysis at the single molecule level [36–39].

Mathematical modeling and model-based data analysis are indispensable for understanding the behavior of biological systems [40,41]. Xu and coworkers [42] proposed a generic rate equation for the dynamical modeling of metabolic kinetics, in which kinetic parameters are clear and precise enough to correlate to experimental data for purposes of constructing a database. The equation is applicable to an arbitrary number of substrates and products for computation of dynamic modeling, which makes the construction of omics scale networks using kinetic models much more feasible.

Because enzymes are the central components of biological networks, any change related to an enzyme, even a slight fluctuation in enzyme content or activity, will lead to local or global alteration of biological networks. For example, CD38 is an ADP-ribosyl cyclase responsible for synthesizing cADPR and NAADP. Through these two secondary messengers, CD38 can regulate insulin secretion, susceptibility to bacterial infection, and even social behavior of mice (see the review by Lee [43]). Therefore, enzymes can be used as markers to characterize the status of biological systems and trace the dynamic changes of biological networks. Identification of highly specific and sensitive enzyme markers from biological networks for various purposes has been a major task for enzymologists. Schizophrenia is a common and severe neuropsychiatric disorder. Although many scientists believe that increased oxidative stress is relevant to the pathophysiology of schizophrenia, it is still debated whether oxidoreductases could be used as biomarkers for diagnosis and treatment of this disease [44]. In a recent research article, Zhang and coworkers [45] performed a meta-analysis of data from early studies related to oxidative stress in schizophrenia, and found that superoxide dismutase (SOD) activity significantly decreased in the disorganized type of schizophrenia patients, indicating that SOD may be a potential biomarker of schizophrenia. Their results provide an exciting opportunity for diagnosis and treatment of schizophrenia.

Thanks to intensive research efforts from an increasing number of laboratories, detailed information regarding the physics, chemistry and kinetics of enzymes is accumulating rapidly. However, theoretical developments in this area have lagged behind experimental data. Significant evidence shows that in some cases, the observed behaviors of enzymes are incompatible with the classical theories of enzymatic catalysis. For example, when biologists began to study the dynamic properties of biological networks, the possible role of protein motion in enzyme catalysis was still controversial [46,47]. Therefore, elucidating the mechanisms of enzymatic catalysis remains one of the most intriguing biochemical phenomena. Zhang and coworkers [48] investigated the effect of substrate-induced conformation changes on enzyme catalysis, and found that the activity and conformation of an enzyme is changed when the enzyme induced with one substrate was reacted with other substrates in subsequent reactions. Based on their results, the authors ingeniously brought the two classical catalysis models, "induced fit" and "lock and key" together, and proposed a new model called "induced fit-lock and key" for enzyme action, potentially deepening our understanding of enzyme catalysis.

One of the major goals of enzymology is to develop efficient biocatalytic systems for industrial, medical or environmental applications. Along with the rapidly growing understanding of enzyme action in the context of biological systems, our capacity to design biocatalytic systems is growing at an unprecedented pace. While the early studies of biocatalysis were restricted to "one-step" biotransformations or optimization of an organism's own metabolic pathways, we are now able to engineer entire foreign biosynthetic pathways into host organisms to make non-native natural or even unnatural products [49,50]. However, biocatalysis still has many challenges that need to be addressed, most of which are around enzyme discovery, design, and optimization. For example, how can we find or create enzymes that catalyze the desired chemical reaction of interest, even those not existing in nature? How can we create multiple-enzyme biocatalytic systems required for multistep biosynthesis of complex natural and unnatural products? How can we effectively integrate non-native pathways into the metabolism of a host? How can we improve the stress tolerance of biocatalytic systems to industrial reaction conditions? The following papers will be helpful for biologists to answer these questions.

The identification and isolation of novel enzymes is an extremely active field in enzymology. There are two general experimental strategies for enzyme discovery, which are based on function (activity) or sequence, respectively. The function-based strategy is a straightforward way to obtain enzymes which have desired functions. For example, using molecular probes that bind to enzymes of interest in an activity-dependent manner, enzymologists not only can quantify the dynamics of an enzyme *in vivo*, but also can directly isolate and identify the enzyme with the desired function from cell or tissue samples [51]. This technique, termed activity-based protein profiling (ABPP), is being widely applied to the discovery of novel enzymes and drugs [52,53]. Compared with the function-based strategy, the sequence-based strategy is readily performed using bioinformatics and PCR approaches, and is widely used in many laboratories. Using the sequence-based strategy, Sun and Liu [54] successfully isolated a new carboxylesterase gene from *Geobacillus stearothermophilus*. Because of the fact that the enzyme exhibited a high thermal stability and broad pH tolerance, the authors suggested that this carboxylesterase had potential applications in the synthesis of stereospecific compounds and degradation of environmental contaminants. Although these two experimental strategies have been very successful in enzyme discovery, biological experimentation alone is clearly inadequate to meet the challenge presented by the exponentially increasing number of unknown sequences. To address this challenge, a large-scale collaborative project, the Enzyme Function Initiative (EFI), was established in 2010. The EFI is developing a computation-based multidisciplinary strategy for functional assignment of unknown enzymes in a high throughput manner [55].

While more and more novel enzymes are identified from diverse organisms, especially those in extreme environments, a new challenge for applied enzymologists is how to assign each enzyme to the job that suits it best. For example, early studies showed that the (R)-oxynitrilase from *Prunus salicina* was one of the poorest oxynitrilases for the synthesis of (R)-cyanohydrins from hydrogen cyanide with aldehydes, and thereby not suitable for industrial applications. However, in a recent research article, Liu and Zong [56] reported that plum (R)-oxynitrilase could efficiently catalyze enantioselective transcyanation of acetyltrimethylsilane with acetone cyanohydrin, and both acetyltrimethylsilane conversion and enantiomeric excess of the product were above 99%. This finding implies that identifying new catalytic functions of known enzymes is an efficient way to increase the potential applications of enzymes in biotechnology. Although enzymes are usually regarded as specific catalysts of biochemical reactions, the promiscuity of some enzymes is a common phenomenon, and the structural, mechanistic, and evolutionary implications of this were discussed in a recent review [57]. Considering the fact that industrial reaction conditions are different from physiological conditions, the secondary capabilities of some enzymes may make them more suitable for industrial applications in certain cases.

The interest in utilizing enzymes in industrial processes is constantly growing during the past several decades. However, natural enzymes are usually not optimally suited for industrial applications because they have evolved to work in the mild cellular environment rather than under harsh process conditions. Fortunately, many strategies have been developed to overcome this problem, the most popular ones of which are directed evolution, rational design, chemical modification and immobilization. Using these approaches, many biocatalysts with excellent properties were developed and used in various applications. For example, Wu and coworkers [58] developed a novel capillary trypsin reactor by organic-inorganic hybrid monolith based immobilization. This trypsin reactor not only has high enzymatic activity, but also shows reliable mechanical stability and permeability. This reactor is promising for the development of an automated online digestion device for use in proteomics. The recent advances, new challenges and future trends of directed evolution, rational design, chemical modification and immobilization are comprehensively discussed in a number of reviews [59–63].

One of the major technological hurdles in enzymology research is how to control the expression of the enzyme of interest at the desired level. Although many biologists hope to maximize the expression yield, some enzymologists who engage in metabolic engineering and synthetic biology are facing a greater challenge: how to optimize the absolute and relative levels of the enzyme components of the designed biosynthetic pathway, and how to balance the activities of endogenous and exogenous enzymes. For example, to make a Taxol precursor in *Escherichia coli*, the expression level of more than 30 endogenous and exogenous enzymes were optimized [64]. Therefore, the development of novel expression strategies has an important impact on enzymological research. Two recent research articles introduced two new enzyme expression strategies. A functional fragment of coagulation factor VIII (BDD-FVIII) has potential for use in the therapy of hemophilia A. However, because of its large size, it is difficult to deliver the BDD-FVIII gene into cells by a single adeno-associated virus (AAV) vector, slowing the development of gene therapy for hemophilia A. Zhu and coworkers [65] presented a protein trans-splicing based dual-vector delivery system which is suitable for the expression of larger proteins in mammalian cells. Using this delivery system, BDD-FVIII was successfully expressed in HEK-293 and COS-7 cells. These results not only pave the way for the development of gene therapy for hemophilia A, but also offer a new strategy for the expression of large foreign proteins. Wang *et al.* [66] inserted *H. polymorpha*-derived ribosomal DNA (rDNA)-targeting element into a pPIC9K-derived expression vector containing the pGAP promoter, and developed a high-efficiency *H. polymorpha* expression system, by which the yield of recombinant T4 lysozyme was up to 0.5 g $L^{-1}$. Besides describing a high-efficiency expression system for production of heterologous protein, their results also provide a strategy for the construction of a high-efficiency expression system.

Beyond the studies of "one-step" biotransformations, the exploration and development of multiple-enzyme biocatalytic systems is of great interest and is an important challenge to scientists. In past years, researchers have successfully engineered and constructed many important multiple-enzyme biocatalytic systems *in vivo* and *in vitro* [67,68], some of which are even composed of tens of enzyme components. For example, cellulosome is a multi-enzyme complex from anaerobic microorganisms which consists of a large number of enzymatic components arranged around non-enzymatic scaffolding proteins. Because of its potential biotechnological applications in the conversion of intractable cellulosic biomass into sugars, cellulosome is of wide interest in enzymology and biotechnology. In a recent review, Wang *et al.* [69] summarized the latest advances and future challenges in the reconstitution of cellulosome. Besides describing the structure and assembly mode of cellulosome, the authors introduced a general strategy for reengineering cellulosome which also could be applied in the design and development of other multiple-enzyme complexes.

At the end of the 20th century, many people predicted that enzymology as a mature field of research over one century would inevitably decline in importance. However, because of the development of new techniques and theories, and the need for sustainable industrial development, the scope of enzymology has unexpectedly been expanding rapidly in the past decade. For example, under the idea of systems, enzymology is robustly being integrated with all areas of biology and other related disciplines, as discussed in the articles mentioned above and other reviews elsewhere. With the rapid evolution of enzymology from a traditional science which focused on simple enzymological characterization to the cross-discipline, high-content modern form, more and more scientists believe that enzymology not only has a booming present, but also will have a bright future.

*This work was supported in part by the National Natural Science Foundation of China (Grant No. 30800548) and the Project Sponsored by the Scientific Research Foundation for the Returned Overseas Chinese Scholars, Ministry of Education of China.*

**Open Access** This article is distributed under the terms of the Creative Commons Attribution License which permits any use, distribution, and reproduction in any medium, provided the original author(s) and source are credited.

Contributed equally to this work

This article is published with open access at Springerlink.com


References

1 Michaelis L. Menten M. L. Die Kinetik der Invertinwirkung Biochem Z 1913 49 333 369
2 Zalatan J. G. Herschlag D. The far reaches of enzymology Nat Chem Biol 2009 5 516 520 10.1038/nchembio0809-516 19620986
3 Derouiche A, Cousin C, Mijakovic I. Protein phosphorylation from the perspective of systems biology. Curr Opin Biotechnol, 2011, 24, doi: 10.1016/j.copbio.2011.11.00810.1016/j.copbio.2011.11.008 22119098
4 Han Y. F. Yang Q. Zhang S. W. Receptor-like kinase CrRLK1-L subfamily: novel motifs in extracellular domain and biological functions in plants Prog Biochem Biophys 2011 38 891 899 10.3724/SP.J.1206.2011.00085
5 Tu L. H. Liu H. P. Luo J. A new family of regulators of calcineurin (RCANs) Prog Biochem Biophys 2010 37 22 28 10.3724/SP.J.1206.2009.00287
6 Ding S. H. Yin X. M. Shi J. H. GSK-3β modulates 9G8-mediated alternative splicing of tau exon 10 Prog Biochem Biophys 2010 37 161 166 10.3724/SP.J.1206.2009.00528
7 Xie M. Chen Q. C. Liao X. M. The protective effects of ubiquitin C-terminal hydrolase L1 on neurons Prog Biochem Biophys 2010 37 1054 1058 10.3724/SP.J.1206.2010.00253
8 Sun L. Yang Y. D. Liu D. B. Deubiquitinase activity and regulation of antiviral innate immune responses by papain-like proteases of human coronavirus NL63 Prog Biochem Biophys 2010 37 871 880 10.3724/SP.J.1206.2010.00111
9 Wang C. J. Lin J. Zhang J. J. Progress in the study of prokaryotic ubiquitin-like protein (Pup)-proteasome system Prog Biochem Biophys 2011 38 1091 1098 10.3724/SP.J.1206.2011.00110
10 Guo X. Q. The function of PRDM9 on mammalian recombination hotspots Prog Biochem Biophys 2010 37 929 931 10.3724/SP.J.1206.2010.00224
11 Peng Q. Chen W. C. Liu X. G. Advances in relationship between deacetylase (Sirtuin) and aging Prog Biochem Biophys 2010 37 1271 1277 10.3724/SP.J.1206.2010.00241
12 Zhang Y. J. Jiang J. H. Xie J. Lysyl oxidases related to human diseases Prog Biochem Biophys 2011 38 389 399 10.3724/SP.J.1206.2010.00468
13 Chen J. W. Zuo Q. H. Ji L. The research advances in ubiquitin-independent degradation of proteins Prog Biochem Biophys 2011 38 593 603 10.3724/SP.J.1206.2010.00569
14 Wu W. S. Zheng X. Duan X. L. Transmembrane serine proteases 6: a newly discovered hepcidin regulator Prog Biochem Biophys 2010 37 235 238 10.3724/SP.J.1206.2009.00584
15 Mason S. D. Joyce J. A. Proteolytic networks in cancer Trends Cell Biol 2011 21 228 237 10.1016/j.tcb.2010.12.002 21232958 PMC3840715
16 Tian X. Li C. Li Y. Y. Analysis of inhibitory activity and antineoplastic effect of wild type rBTI and its mutants Prog Biochem Biophys 2010 37 654 661 10.3724/SP.J.1206.2009.00731
17 Lu Y. Luo F. Yang M. Suppression of glutamate synthase genes significantly affects carbon and nitrogen metabolism in rice (Oryza sativa L.) Sci China Life Sci 2011 54 651 663 10.1007/s11427-011-4191-9 21748588
18 Sun S. N. Gui Y. H. Jiang Q. The effects of dihydrofolate reductase gene on the development of pharyngeal arches Prog Biochem Biophys 2010 37 145 153 10.3724/SP.J.1206.2009.00409
19 Li B. Zhang D. Zeng J. A mutation of testis-specific lactate dehydrogenase gene found in male patients with unexplained infertility Prog Biochem Biophys 2010 37 445 450 10.3724/SP.J.1206.2009.00526
20 Heinemann M. Sauer U. Systems biology of microbial metabolism Curr Opin Microbiol 2010 13 337 343 10.1016/j.mib.2010.02.005 20219420
21 Gerosa L. Sauer U. Regulation and control of metabolic fluxes in microbes Curr Opin Biotechnol 2011 22 566 575 10.1016/j.copbio.2011.04.016 21600757
22 Ginsberg G. Guyton K. Johns D. Genetic polymorphism in metabolism and host defense enzymes: implications for human health risk assessment Crit Rev Toxicol 2010 40 575 619 10.3109/10408441003742895 20662711
23 Hao D. Xiao P. Chen S. Phenotype prediction of nonsynonymous single nucleotide polymorphisms in human phase II drug/xenobiotic metabolizing enzymes: perspectives on molecular evolution Sci China Life Sci 2010 53 1252 1262 10.1007/s11427-010-4062-9 20953949
24 Luo H. Chen X. Ding M. Study on the mechanism of effects of lomefloxacin on biological properties of bloom syndrome helicase Prog Biochem Biophys 2011 38 1060 1071 10.3724/SP.J.1206.2011.00178
25 Hu J. P. Liu W. Tang D. Y. Study on the binding mode and mobility of HIV-1 integrase with L708, 906 inhibitor Prog Biochem Biophys 2011 38 338 346 10.3724/SP.J.1206.2010.00438
26 He H. Liu B. Zhang X. Development of a high-throughput assay for the HIV-1 integrase disintegration reaction Sci China Life Sci 2010 53 241 247 10.1007/s11427-010-0006-7 20596834
27 Lu L. N. Tang T. S. Guo C. X. Advances of study on translesion DNA synthesis polymerase kappa in mammalian cells Prog Biochem Biophys 2011 38 204 209 10.3724/SP.J.1206.2010.00533
28 Fan S. H. Shan L. W. Wang B. L. Preparation and characterization of T7 endonuclease I with single active domain Prog Biochem Biophys 2010 37 426 432 10.3724/SP.J.1206.2009.00580
29 Martínez-Espinosa R. M. Cole J. A. Richardson D. J. Enzymology and ecology of the nitrogen cycle Biochem Soc Trans 2011 39 175 178 10.1042/BST0390175 21265768
30 Weedon J. T. Aerts R. Kowalchuk G. A. Enzymology under global change: organic nitrogen turnover in alpine and sub-Arctic soils Biochem Soc Trans 2011 39 309 314 10.1042/BST0390309 21265794
31 Zhang H. Huang Y. Ye X. Analysis of the contribution of acid phosphatase to P efficiency in Brassica napus under low phosphorus conditions Sci China Life Sci 2010 53 709 717 10.1007/s11427-010-4008-2 20602274
32 Guo J. Wu G. Wan F. Temporal allocation of metabolic tolerance to transgenic Bt cotton in beet armyworm, Spodoptera exigua (Hübner) Sci China Life Sci 2011 54 152 158 10.1007/s11427-010-4133-y 21318485
33 Grassmann J. Scheerle R. K. Letzel T. Functional proteomics: application of mass spectrometry to the study of enzymology in complex mixtures Anal Bioanal Chem 2012 402 625 645 10.1007/s00216-011-5236-4 21769551
34 Van Noorden C. J. Imaging enzymes at work: metabolic mapping by enzyme histochemistry J Histochem Cytochem 2010 58 481 497 10.1369/jhc.2010.955518 20124092 PMC2874181
35 Kovarik M. L. Allbritton N. L. Measuring enzyme activity in single cells Trends Biotechnol 2011 29 222 230 10.1016/j.tibtech.2011.01.003 21316781 PMC3080453
36 van Oijen A. M. Single-molecule approaches to characterizing kinetics of biomolecular interactions Curr Opin Biotechnol 2011 22 75 80 10.1016/j.copbio.2010.10.002 21036593
37 Claessen V. I. Engelkamp H. Christianen P. C. Single-biomolecule kinetics: the art of studying a single enzyme Annu Rev Anal Chem 2010 3 319 340 10.1146/annurev.anchem.111808.073638 20636045
38 Chen Q. Groote R. Schönherr H. Probing single enzyme kinetics in real-time Chem Soc Rev 2009 38 2671 2683 10.1039/b903638e 19690746
39 Gershenson A. Single molecule enzymology: watching the reaction Curr Opin Chem Biol 2009 13 436 442 10.1016/j.cbpa.2009.06.011 19632886
40 Chen W. W. Niepel M. Sorger P. K. Classic and contemporary approaches to modeling biochemical reactions Genes Dev 2010 24 1861 1875 10.1101/gad.1945410 20810646 PMC2932968
41 Cloots L. Marchal K. Network-based functional modeling of genomics, transcriptomics and metabolism in bacteria Curr Opin Microbiol 2011 14 599 607 10.1016/j.mib.2011.09.003 21943683
42 Xu M. J. Zhu X. M. Lin B. H. Generic enzymatic rate equation Prog Biochem Biophys 2011 38 759 767 10.3724/SP.J.1206.2010.00500
43 Lee H. C. Cyclic ADP-ribose and NAADP: fraternal twin messengers for calcium signaling Sci China Life Sci 2011 54 699 711 10.1007/s11427-011-4197-3 21786193
44 Bitanihirwe B. K. Woo T. U. Oxidative stress in schizophrenia: an integrated approach Neurosci Biobehav Rev 2011 35 878 893 10.1016/j.neubiorev.2010.10.008 20974172 PMC3021756
45 Zhang M. Zhao Z. He L. A meta-analysis of oxidative stress markers in schizophrenia Sci China Life Sci 2010 53 112 124 10.1007/s11427-010-0013-8 20596963
46 Nagel Z. D. Klinman J. P. A 21st century revisionist’s view at a turning point in enzymology Nat Chem Biol 2009 5 543 550 10.1038/nchembio.204 19620995
47 McGeagh J. D. Ranaghan K. E. Mulholland A. J. Protein dynamics and enzyme catalysis: insights from simulations Biochim Biophys Acta 2011 1814 1077 1092 10.1016/j.bbapap.2010.12.002 21167324
48 Zhang Z. J. Pan R. Zhou Y. “Induced fit-lock and key” model in enzymic reactions Prog Biochem Biophys 2011 38 418 426 10.3724/SP.J.1206.2011.00052
49 Weber W. Fussenegger M. Emerging biomedical applications of synthetic biology Nat Rev Genet 2011 13 21 35 10.1038/ni.2184 22124480 PMC7097403
50 Lee J. W. Na D. Park J. M. Systems metabolic engineering of microorganisms for natural and non-natural chemicals Nat Chem Biol 2012 8 536 546 10.1038/nchembio.970 22596205
51 Heal W. P. Dang T. H. Tate E. W. Activity-based probes: discovering new biology and new drug targets Chem Soc Rev 2011 40 246 257 10.1039/C0CS00004C 20886146
52 Nomura D. K. Dix M. M. Cravatt B. F. Activity-based protein profiling for biochemical pathway discovery in cancer Nat Rev Cancer 2010 10 630 638 10.1038/nrc2901 20703252 PMC3021511
53 Chang J. W. Nomura D. K. Cravatt B. F. A potent and selective inhibitor of KIAA1363/AADACL1 that impairs prostate cancer pathogenesis Chem Biol 2011 18 476 484 10.1016/j.chembiol.2011.02.008 21513884 PMC3119342
54 Sun J. X. Liu Z. B. Heterologous expression and characterization of the carboxylesterase from Geobacillus stearothermophilus Prog Biochem Biophys 2010 37 967 974 10.3724/SP.J.1206.2010.00134
55 Gerlt J. A. Allen K. N. Almo S. C. The enzyme function initiative Biochemistry 2011 50 9950 9962 10.1021/bi201312u 21999478 PMC3238057
56 Liu S. L. Zong M. H. (R)-oxynitrilase from Prunus salicina catalysed synthesis of (R)-ketone-cyanohydrin by enantioselective transcyanation Prog Biochem Biophys 2010 37 1212 1216 10.3724/SP.J.1206.2010.00147
57 Khersonsky O. Tawfik D. S. Enzyme promiscuity: a mechanistic and evolutionary perspective Annu Rev Biochem 2010 79 471 505 10.1146/annurev-biochem-030409-143718 20235827
58 Wu S. Ma J. Yang K. A novel organic-inorganic hybrid monolith for trypsin immobilization Sci China Life Sci 2011 54 54 59 10.1007/s11427-010-4108-z 21253871
59 Brady D. Jordaan J. Advances in enzyme immobilisation Biotechnol Lett 2009 31 1639 1650 10.1007/s10529-009-0076-4 19590826
60 Dalby P. A. Strategy and success for the directed evolution of enzymes Curr Opin Struct Biol 2011 21 473 480 10.1016/j.sbi.2011.05.003 21684150
61 Diaz-Rodriguez A. Davis B. G. Chemical modification in the creation of novel biocatalysts Curr Opin Chem Biol 2011 15 211 219 10.1016/j.cbpa.2010.12.002 21276746
62 Otten L. G. Hollmann F. Arends I. W. Enzyme engineering for enantioselectivity: from trial-and-error to rational design? Trends Biotechnol 2010 28 46 54 10.1016/j.tibtech.2009.10.001 19913316
63 Saven J. G. Computational protein design: engineering molecular diversity, nonnatural enzymes, nonbiological cofactor complexes, and membrane proteins Curr Opin Chem Biol 2011 15 452 457 10.1016/j.cbpa.2011.03.014 21493122 PMC4077665
64 Ajikumar P. K. Xiao W. H. Tyo K. E. Isoprenoid pathway optimization for Taxol precursor overproduction in Escherichia coli Science 2010 330 70 74 10.1126/science.1191652 20929806 PMC3034138
65 Zhu F. Liu Z. Chi X. Protein trans-splicing based dual-vector delivery of the coagulation factor VIII gene Sci China Life Sci 2010 53 683 689 10.1007/s11427-010-4011-7 20602271
66 Wang N. Wang Y. Li G. Expression, characterization, and antimicrobial ability of T4 lysozyme from methylotrophic yeast Hansenula polymorpha A16 Sci China Life Sci 2011 54 520 526 10.1007/s11427-011-4174-x 21706412
67 Kwon S. J. Mora-Pale M. Lee M. Y. Expanding nature’s small molecule diversity via in vitro biosynthetic pathway engineering Curr Opin Chem Biol 2012 16 186 195 10.1016/j.cbpa.2012.02.001 22397884
68 Wong F. T. Khosla C. Combinatorial biosynthesis of polyketides—a perspective Curr Opin Chem Biol 2012 16 117 123 10.1016/j.cbpa.2012.01.018 22342766 PMC3328608
69 Wang J. L. Wang L. S. Liu W. F. Research advances on the assembly mode of cellulosomal macromolecular complexes Prog Biochem Biophys 2011 38 28 35 10.3724/SP.J.1206.2010.00278
